# Supplementary material for: Hematological diseases-related mucormycosis: A retrospective single center study
Source: PLoS Negl Trop Dis. 2025 Sep 26;19(9):e0013531. doi: 10.1371/journal.pntd.0013531 (PMC12468762; doi:10.1371/journal.pntd.0013531)
Supplement: Attachment — Submitted filename: Response to Reviewers.docx [file pntd.0013531.s004.docx]

Dear Editor，

We thank the reviewers, associate editor and you for the positive and very constructive comments on our manuscript entitled “Hematological diseases-Related Mucormycosis: A Retrospective Single Center Study” (PNTD-D-25-00614). These incisive critiques are extremely helpful in guiding us to improve the quality of this manuscript.

In the revised manuscript, we have made improvements in response to the reviewers’ and editors’ comments; please see the attached response following this letter. We greatly appreciate any further suggestions regarding this manuscript.

With Best Regards,

Xudong wei, Ph.D.

-----------------------------------------------------------------

Director and Professor

Department of Hematology

Cancer Hospital Affiliated to Zhengzhou University

Zhengzhou, Henan, China. 450008

E-mail: [xudongwei@zzu.edu.cn](mailto:xudongwei@zzu.edu.cn)

**Methods**

**Reviewer #1:** Line 69: was culture and mNGS performed on all patients included, if not what percentage had either and both?
Additionally, the concordance rate for microbiologic confirmed infection would also be of interest to the reader.

Thank so much for your professional reviews. Yes, this study analyzed 46 patients suspected of having hematological malignancies complicated by mucormycosis. All patients underwent culture and high - throughput sequencing. We have provided additional explanations in the "Patients" section of the methods part in the revised manuscript.

Thanks for your good comment. We have made supplementary additions to the first paragraph of the Results section and the second paragraph of the Discussion section in the revised manuscript:

In this study, the concordance rate between traditional cultivation methods and metagenomic next-generation sequencing (mNGS) detection was found to be 19.57%. This finding aligns with previous research[1]. The primary reasons for this discrepancy include the inherent biological characteristics of mucormycosis, which are not conducive to in vitro culture, as well as limitations in sample processing that result in challenges in cultivation and a low positive rate. In contrast, mNGS is a culture-independent approach for the identification of pathogenic microorganisms. It employs high-throughput sequencing technology to detect and characterize microbial DNA and/or RNA in patient samples, subsequently comparing and analyzing these sequences against a comprehensive database. This allows for the unbiased detection of a wide range of infections within the samples[2]. mNGS offers significant advantages, particularly for immunocompromised individuals and patients whose blood culture positivity rates are diminished due to the administration of potent antibiotics. Currently, mNGS is recommended as a first-line diagnostic tool for patients undergoing hematopoietic stem cell transplantation and solid organ transplantation or as an adjunctive diagnostic method[3, 4].

Reviewer #2: Methods appear valid.

Thanks so much for your positive comments.

Reviewer #3:

1) objectives are clearly stated and the study design matches the objectives

2)population is clearly describes.

3) as far as sample size is concerned, with only 46 patients, subgroup analyses (e.g., multivariate Cox regression for multiple variables) may be underpowered, increasing the risk of type II error. However, given the rarity of hematologic mucormycosis, the sample size is reasonable for a single-center retrospective study, though this limitation should be more explicitly discussed.

4) statistical analyses used is also appropriate

4) yes concerns regarding ethical or regulatory requirements are met

Thanks for your good comment. We fully agree with your viewpoint and have added the following supplementary explanation in the "Discussion" section of the revised draft:

This study is a single-center, retrospective case analysis. The sample size is small, and there may be potential selection bias, which brings certain limitations. Therefore, in future research, we plan to conduct a larger-scale, multi-center study.

**Results**

Reviewer #1: Given the focus on COVID-19 and the impact on steroid use and thereby assumption of the correlation with Mucor rate of rise, it would be informative to capture the frequency of patient flagged for steroid exposure who also had concomitant COVID-19 diagnosed.

Thank so much for your professional reviews. Due to national policy constraints during the COVID-19 pandemic, we were unable to evaluate the association between COVID-19 infection and steroid hormone use among the patients included in this study. Nevertheless, in the third-to-last paragraph of the "Discussion" section of the revised manuscript, we have included supplementary information on related research：

Since 2021, the incidence of COVID-19-associated mucormycosis has been on the rise. Systemic corticosteroid therapy, while reducing mortality in severe COVID-19 cases, increases the risk of secondary mycosis[5]. Corticosteroids are indicated for hospitalized patients with COVID-19 pneumonia who require supplemental oxygen and advanced respiratory support[5]. However, there is a prevalent inappropriate and excessive use of corticosteroids in the management of COVID-19, particularly in outpatient settings[6]. The administration of corticosteroids in COVID-19 cases can elevate the risk of immunosuppression[7], including alterations in macrophage and neutrophil function, insulin resistance, and increased blood sugar[8, 9]

Figure 1 cold benefit from alternative data presentation such as a heatmap.

This is a nice comment. We have already included the heat map in Figure 1B. The pie chart has the advantage of effectively reflecting various proportions, while the heat map offers a relatively concise and simple presentation.

Reviewer #2: Analysis presented matches the analysis plan, and the results are clearly presented.

Thanks so much for your positive comments.

Reviewer #3: 1) Table 1 (Mucor strain distribution) and Table 2 (patient characteristics) are useful and well-structured, though Table 1 includes redundant rows (e.g., mixed strain listings that are unclear) — this could confuse readers and needs cleanup.
2) Table 3 (Cox analysis) is detailed and informative, but there are formatting inconsistencies — e.g., irregular column alignment and missing confidence intervals in some entries.
3) Consider expanding discussion on treatment efficacy outcomes to provide more clinical interpretation.

Thanks for your good comment.

1) We further verified the names of the bacteria and made standardized modifications. Regarding the issue of redundant lines, we corrected the expression to indicate that this sample was a mixed infection involving multiple bacteria.

2) In Table 3, pertaining to the Cox proportional hazards analysis, we incorporated variables with a p-value of less than 0.05 from the univariate analysis into the multivariate analysis. These variables include age ≥ 60, presence of other comorbidities, administration of high-dose corticosteroids, occurrence of two or more Mucor infections, and a decrease in neutrophils for more than 10 days. Variables not meeting this criterion(Univariate analysis: P < 0.05) were excluded from the multivariate analysis, and consequently, 95% confidence intervals are not provided for them(excluded from the multivariate analysis). Should it be deemed necessary, we are prepared to list the positive indicators separately and furnish the negative indicators as supplementary material.

3) Thank so much for your professional reviews. We have provided additional explanations in the fourth paragraph of the discussion section of the revised version:

Currently, monotherapy is commonly employed. First-line monotherapy options include liposomal AmB, isavuconazole, and posaconazole. Liposomal AmB is strongly recommended, and for salvage treatment, the use of two triazole drugs is highly recommended[10]. Research has demonstrated that there is no significant difference in the therapeutic efficacy between the use of amphotericin B liposomes alone and their combination with posaconazole[11], aligning with our findings. Conversely, some studies suggest that combining isavuconazole or posaconazole with amphotericin B liposomes is more effective than using amphotericin B liposomes alone[12, 13]. Previous research has indicated no significant difference in the therapeutic effects of antifungal drugs for mucormycosis compared to amphotericin B treatment[14], which is consistent with our results. However, other studies have reported that amphotericin B exhibits a stronger therapeutic effect on mucormycosis than combined antifungal drugs[12, 13]. It is important to note that this study is a single-center, small-sample retrospective analysis, which presents certain limitations. Consequently, larger-scale, multi-center studies with more systematic evaluations are necessary in the future.

**Conclusions**

Reviewer #1: The authors provide convincing analysis of the regional drivers of Mucor infection in hematologic patients, noting high dose steroid exposure, advanced age, and multiple Mucor infections as clear drivers of increased mortality risk. As noted above, commentary about COVID-19 is made without evidence of infection in captured patient base, this may be related to regional access to testing at this time, but if testing was available as a standard of care, commentary on the association of this diagnosis with steroid exposure in these patients would be of interest to support the complications offered in the conclusion.

Thank so much for your professional reviews. Due to national policy constraints during the COVID-19 pandemic, we were unable to evaluate the association between COVID-19 infection and steroid hormone use among the patients included in this study. Nevertheless, in the third-to-last paragraph of the "Discussion" section of the revised manuscript, we have included supplementary information on related research：

Since 2021, the incidence of COVID-19-associated mucormycosis has been on the rise. Systemic corticosteroid therapy, while reducing mortality in severe COVID-19 cases, increases the risk of secondary mycosis[5]. Corticosteroids are indicated for hospitalized patients with COVID-19 pneumonia who require supplemental oxygen and advanced respiratory support[5]. However, there is a prevalent inappropriate and excessive use of corticosteroids in the management of COVID-19, particularly in outpatient settings[6]. The administration of corticosteroids in COVID-19 cases can elevate the risk of immunosuppression[7], including alterations in macrophage and neutrophil function, insulin resistance, and increased blood sugar[8, 9]

Reviewer #2: The conclusions are justified and supported by the data.

Thanks so much for your positive comments.

Reviewer #3: The discussion lightly touches on some known limitations — e.g., challenges in histopathological diagnosis, limitations of traditional culture methods, and the value of mNGS in such contexts.
However, key limitations are not explicitly acknowledged, such as:
Small sample size (n=46) and its implications for statistical power.
Single-center design and retrospective nature, which may limit generalizability.
Potential selection bias (e.g., inclusion only of patients who underwent mNGS).

Thanks for your good comment. We fully agree with your viewpoint and have added the following supplementary explanation in the "Discussion" section of the revised draft:

This study is a single-center, retrospective case analysis. The sample size is small, and there may be potential selection bias, which brings certain limitations. Therefore, in future research, we plan to conduct a larger-scale, multi-center study.

**Editorial and Data Presentation Modifications?**

Reviewer #1: I would encourage the authors to define nGS for the reader within the manuscript with first reference as well as in the abstract.
line 319 potentially unintended space between Mucor and mycosis.
Would review Table for unnecessary periods, and consider standard italicized nomenclature in the manuscript where appropriate
Figure 2 grpahic appears blurry in my editorial review sample. Would provide high resolution alternative.

Thank so much for your professional reviews. We have supplemented the definition of mNGS in the abstract and first paragraph of the introduction section:

Metagenomic next-generation sequencing (mNGS) refers to the application of high-throughput sequencing technology for the comprehensive analysis of nucleic acid content in patient samples, facilitating the detection and characterization of microbial DNA and/or RNA, and then comparing and analyzing the results with an information database to determine the types of pathogenic microorganisms present in the sample.[2].

Thank you for pointing out our mistakes. We have removed the incorrect spaces, adopted the correct standard italicized nomenclature, and replaced them with higher-resolution images.

Reviewer #2: (No Response)

Reviewer #3: (No Response)

**Summary and General Comments**

Reviewer #1: The authors provide a regional assessment of Mucor infection in hematologic patients with descriptive statistical approach to the demographics and epidemiology of invasive mucor. The key points made appear supported by the data offered. The manuscript could be strengthened by commentary on concordance rates where standard microbiology techniques and nGS were performed on the same patient to allow for assessment of the non-commercial nGS approach described as it relates to "traditional" mycology workflows.

Thank so much for your professional reviews. this study analyzed 46 patients suspected of having hematological malignancies complicated by mucormycosis. All patients underwent culture and high - throughput sequencing. We have provided additional explanations in the "Patients" section of the methods part in the revised manuscript.

Thanks for your good comment. We have made supplementary additions to the first paragraph of the Results section and the second paragraph of the Discussion section in the revised manuscript:

In this study, the concordance rate between traditional cultivation methods and metagenomic next-generation sequencing (mNGS) detection was found to be 19.57%. This finding aligns with previous research[1]. The primary reasons for this discrepancy include the inherent biological characteristics of mucormycosis, which are not conducive to in vitro culture, as well as limitations in sample processing that result in challenges in cultivation and a low positive rate. In contrast, mNGS is a culture-independent approach for the identification of pathogenic microorganisms. It employs high-throughput sequencing technology to detect and characterize microbial DNA and/or RNA in patient samples, subsequently comparing and analyzing these sequences against a comprehensive database. This allows for the unbiased detection of a wide range of infections within the samples[2]. mNGS offers significant advantages, particularly for immunocompromised individuals and patients whose blood culture positivity rates are diminished due to the administration of potent antibiotics. Currently, mNGS is recommended as a first-line diagnostic tool for patients undergoing hematopoietic stem cell transplantation and solid organ transplantation or as an adjunctive diagnostic method[3, 4].

Reviewer #2: The paper is a retrospective review that describes the clinical characteristics of 46 cases of Mucormycosis diagnosed at the Affiliated Cancer Hospital of Zhengzhou. Using regression analyses, the authors conclude that age greater than 60 years old, prolonged neutropenia (> 10 days), and having two or more Mucor infections were independent risk factors for reduced survival due to Mucormycosis.
While the findings of the paper appear valid, the overall sample size for Mucormycosis is somewhat small and the findings reinforce what is already generally well recognized for infections with fungi in the order Mucorales.

Thanks for your good comment.

Reviewer #3: (No Response)

1.	Yang H, Xu N, Yan M, Yang L, Wen S, Wang S, et al. Comparison of metagenomic next-generation sequencing and conventional culture for the diagnostic performance in febrile patients with suspected infections. BMC infectious diseases. 2024;24(1):350. Epub 2024/03/27. doi: 10.1186/s12879-024-09236-w. PubMed PMID: 38532348; PubMed Central PMCID: PMCPMC10964532.

2.	Miller S, Chiu C. The Role of Metagenomics and Next-Generation Sequencing in Infectious Disease Diagnosis. Clinical chemistry. 2021;68(1):115-24. Epub 2021/12/31. doi: 10.1093/clinchem/hvab173. PubMed PMID: 34969106.

3.	Qian YY, Wang HY, Zhou Y, Zhang HC, Zhu YM, Zhou X, et al. Improving Pulmonary Infection Diagnosis with Metagenomic Next Generation Sequencing. Frontiers in cellular and infection microbiology. 2020;10:567615. Epub 2021/02/16. doi: 10.3389/fcimb.2020.567615. PubMed PMID: 33585263; PubMed Central PMCID: PMCPMC7874146.

4.	Li N, Cai Q, Miao Q, Song Z, Fang Y, Hu B. High-Throughput Metagenomics for Identification of Pathogens in the Clinical Settings. Small methods. 2021;5(1):2000792. Epub 2021/02/23. doi: 10.1002/smtd.202000792. PubMed PMID: 33614906; PubMed Central PMCID: PMCPMC7883231.

5.	Dwivedi S, Choudhary P, Gupta A, Singh S. The cross-talk between mucormycosis, steroids and diabetes mellitus amidst the global contagion of COVID-19. Critical reviews in microbiology. 2023;49(3):318-33. Epub 2022/03/25. doi: 10.1080/1040841x.2022.2052795. PubMed PMID: 35324372.

6.	Ibrahim AS, Gebremariam T, Schwartz JA, Edwards JE, Jr., Spellberg B. Posaconazole mono- or combination therapy for treatment of murine zygomycosis. Antimicrobial agents and chemotherapy. 2009;53(2):772-5. Epub 2008/10/22. doi: 10.1128/aac.01124-08. PubMed PMID: 18936190; PubMed Central PMCID: PMCPMC2630615.

7.	Warkentien T, Rodriguez C, Lloyd B, Wells J, Weintrob A, Dunne JR, et al. Invasive mold infections following combat-related injuries. Clinical infectious diseases : an official publication of the Infectious Diseases Society of America. 2012;55(11):1441-9. Epub 2012/10/09. doi: 10.1093/cid/cis749. PubMed PMID: 23042971; PubMed Central PMCID: PMCPMC3657499.

8.	Rickerts V, Atta J, Herrmann S, Jacobi V, Lambrecht E, Bialek R, et al. Successful treatment of disseminated mucormycosis with a combination of liposomal amphotericin B and posaconazole in a patient with acute myeloid leukaemia. Mycoses. 2006;49 Suppl 1:27-30. Epub 2006/09/12. doi: 10.1111/j.1439-0507.2006.01299.x. PubMed PMID: 16961579.

9.	Luo G, Gebremariam T, Lee H, French SW, Wiederhold NP, Patterson TF, et al. Efficacy of liposomal amphotericin B and posaconazole in intratracheal models of murine mucormycosis. Antimicrobial agents and chemotherapy. 2013;57(7):3340-7. Epub 2013/05/08. doi: 10.1128/aac.00313-13. PubMed PMID: 23650163; PubMed Central PMCID: PMCPMC3697351.

10.	Hoenigl M, Seidel D, Carvalho A, Rudramurthy SM, Arastehfar A, Gangneux JP, et al. The emergence of COVID-19 associated mucormycosis: a review of cases from 18 countries. The Lancet Microbe. 2022;3(7):e543-e52. Epub 2022/02/01. doi: 10.1016/s2666-5247(21)00237-8. PubMed PMID: 35098179; PubMed Central PMCID: PMCPMC8789240 National Institutes of Health. J-PG received speaker and expert advice fees from Pfizer and Gilead. NK has received research grants or honoraria as a speaker or advisor from Astellas, Gilead, MSD, and Pfizer, outside the submitted work. KL received consultancy fees from SMB Laboratories Brussels, MSD, and Gilead; travel support from Pfizer; speaker fees from FUJIFILM Wako, Pfizer, and Gilead; and a service fee from Thermo Fisher Scientific. OAC reports grants or contracts from Amplyx, Basilea, BMBF, Cidara, DZIF, EU-DG RTD (101037867), F2G, Gilead, Matinas, MedPace, MSD, Mundipharma, Octapharma, Pfizer, and Scynexis; Consulting fees from Amplyx, Biocon, Biosys, Cidara, Da Volterra, Gilead, Matinas, MedPace, Menarini, Molecular Partners, MSG-ERC, Noxxon, Octapharma, PSI, Scynexis, and Seres; honoraria for lectures from Abbott, Al-Jazeera Pharmaceuticals, Astellas, Grupo Biotoscana/United Medical/Knight, Hikma, MedScape, MedUpdate, Merck/MSD, Mylan, and Pfizer; payment for expert testimony from Cidara; participation on a data safety monitoring board or advisory board from Actelion, Allecra, Cidara, Entasis, IQVIA, Jannsen, MedPace, Paratek, PSI, and Shionogi; a patent at the German Patent and Trade Mark Office (DE 10 2021 113 0077); and other interests from DGHO, DGI, ECMM, ISHAM, MSG-ERC, and Wiley. JRP received research support from Amplyx, Appili, and Astellas; and honoraria and consulting fees from Pfizer, Appili, and Matinas. PLW has done diagnostic evaluations and received meeting sponsorship from Bruker, Dynamiker, and Launch Diagnostics; speakers fees, expert advice fees, and meeting sponsorship from Gilead; speaker and expert advice fees from F2G; speaker fees MSD and Pfizer; and is a founding member of the European Aspergillus PCR Initiative. ACh received funding support from an educational grant from Pfizer, MSD, and Gilead.

11.	Sen M, Honavar SG, Bansal R, Sengupta S, Rao R, Kim U, et al. Epidemiology, clinical profile, management, and outcome of COVID-19-associated rhino-orbital-cerebral mucormycosis in 2826 patients in India - Collaborative OPAI-IJO Study on Mucormycosis in COVID-19 (COSMIC), Report 1. Indian journal of ophthalmology. 2021;69(7):1670-92. Epub 2021/06/23. doi: 10.4103/ijo.IJO_1565_21. PubMed PMID: 34156034; PubMed Central PMCID: PMCPMC8374756.

12.	Narayanan S, Chua JV, Baddley JW. Coronavirus Disease 2019-Associated Mucormycosis: Risk Factors and Mechanisms of Disease. Clinical infectious diseases : an official publication of the Infectious Diseases Society of America. 2022;74(7):1279-83. Epub 2021/08/23. doi: 10.1093/cid/ciab726. PubMed PMID: 34420052; PubMed Central PMCID: PMCPMC8499811.

13.	Hlaing KM, Monday LM, Nucci M, Nouér SA, Revankar SG. Invasive Fungal Infections Associated with COVID-19. Journal of fungi (Basel, Switzerland). 2023;9(6). Epub 2023/06/27. doi: 10.3390/jof9060667. PubMed PMID: 37367603; PubMed Central PMCID: PMCPMC10303754.

14.	Diamond RD, Haudenschild CC, Erickson NF, 3rd. Monocyte-mediated damage to Rhizopus oryzae hyphae in vitro. Infection and immunity. 1982;38(1):292-7. Epub 1982/10/01. doi: 10.1128/iai.38.1.292-297.1982. PubMed PMID: 7141693; PubMed Central PMCID: PMCPMC347731.
